# Somatostatin Reduces Bile Secretion and Loss of Bile Constituents in Patients with External Biliary Drainage

**DOI:** 10.1155/1996/12097

**Published:** 1996

**Authors:** Helene Bryde Andersen, Ljiljana Petronijevic, Birgitte Giese, Thorkild Mygind, Flemming Burcharth

**Affiliations:** 1 Department of Surgical and Medical Gastroenterology Herlev Hospital University of Copenhagen Denmark; 2 Department of Medical Gastroenterology Herlev Hospital University of Copenhagen Denmark; 3 Department of Radiology Herlev Hospital University of Copenhagen Denmark

## Abstract

The effect of 24-hours continuous somatostatin 14 infusion on the volume of the bile secretion and on the bile composition were studied in seven patients with malignant biliary obstruction who had transhepatic external biliary drainage.

The bile acid composition was measured with high performance liquid chromatography (HPLC). Somatostatin infusion significantly reduced the daily bile loss from median 473 ml to 140 ml (41 percent, p=0.01) with a concomitant significant reduction in the daily molar loss of cholesterol, triglyceride, Na^+^, K^+^, CI^−^, Ca^++^ and Mg^++^. The loss of chloride and sodium was reduced with median 50 mmol/day each (p=0.01). The relative concentrations of the measured bile constituents did not change significantly, except for bile acids (p=0.02): the concentration of glycochenodeoxycholic acid increased significantly (p=0.04). The molar loss of taurocholic acid decreased significantly (p=0.035), so the increased concentration of glycochenodeoxycholic acid resulted only in a marginally significant reduction in the total molar loss of bile acids (p=0.051).

Somatostatin is a potent inhibitor of bile secretion. The peptide may be used in severely bile depleted patients for reducing their serious electrolyte and acidity problems. Analysis of bile acid composition by HPLC is well suited for further investigations of the regulatory mechanisms of bile acid secretion.


*HPB Surgery*, 1996, Vol.9, pp.229–233
Reprints available directly from the publisher
Photocopying permitted by license only

© 1996 OPA (Overseas Publishers Association)
Amsterdam B.V. Published in The Netherlands
by Harwood Academic Publishers GmbH
Printed in Malaysia

### Somatostatin Reduces Bile Secretion and Loss of Bile Constituents in Patients with External Biliary Drainage

HELENE BRYDE ANDERSEN[1], LJILJANA PETRONIJEVIĆ[1], BIRGITTE GIESE[2], THORKILD MYGIND[3] and FLEMMING BURCHARTH[1]

Departments of Surgical[1] and Medical[2] Gastroenterology and Radiology[3], Herlev Hospital, University of Copenhagen, Denmark

*(Received 3 August 1994)*

The effect of 24-hours continuous somatostatin -14 infusion on the volume of the bile secretion and on the bile composition were studied in seven patients with malignant biliary obstruction who had transhepatic external biliary drainage.

The bile acid composition was measured with high performance liquid chromatography (HPLC). Somatostatin infusion significantly reduced the daily bile loss from median 473 ml to 140 ml (41 per cent, $p = 0.01$) with a concomitant significant reduction in the daily molar loss of cholesterol, triglyceride, $Na^+$, $K^+$, $Cl^-$, $Ca^{++}$ and $Mg^{++}$. The loss of chloride and sodium was reduced with median 50 mmol/day each ($p = 0.01$). The relative concentrations of the measured bile constituents did not change significantly, except for bile acids ($p = 0.02$): the concentration of glycochenodeoxycholic acid increased significantly ($p = 0.04$). The molar loss of taurocholic acid decreased significantly ($p = 0.035$), so the increased concentration of glycochenodeoxycholic acid resulted only in a marginally significant reduction in the total molar loss of bile acids ($p = 0.051$).

Somatostatin is a potent inhibitor of bile secretion. The peptide may be used in severely bile depleted patients for reducing their serious electrolyte and acidity problems. Analysis of bile acid composition by HPLC is well suited for further investigations of the regulatory mechanisms of bile acid secretion.

KEY WORDS: Bile acid composition bile secretion chloride cholesterol potassium sodium somatostatin triglyceride external biliary drainage

### INTRODUCTION

Somatostatin is an ubiquitous peptide distributed in the central, peripheral and autonomous nervous system, in the D-cells of the gastrointestinal tract, in the pancreas, and in other peripheral organs [1–4]. It is possible that the peptide exerts its function in several ways: as a paracrine secretion, as a true hormone, and as a neurotransmitter [1–4].Somatostatin and its analogues have many different effects, most of them inhibiting the secretion of hormones and of several gastrointestinal peptides (gastrin, VIP, pancreatic polypeptide, secretin, motilin, insulin, glucagon, growth hormone and thyreotropine)[1,3,4]. Animal experiments and occasional studies in humans have shown that somatostatin reduces bile secretion after a short infusion time[1,4–7]. In man the primary mode of action is on the bile acid-dependent canalicular bile flow, and to some extent on the ductular secretion [2,8]. The effect of somatostatin infusion on bile acids has only been measured after a short infusion period, and no studies have yet reported the specific response on bile acid composition as measured with High Performance Liquid Chromatography (HPLC) [9,10].

The aim of the present study was to evaluate the 24 hour effect of continuous somatostatin-14 infusion on bile secretion, and on bile composition including the

*Correspondence to*: Helene Bryde Andersen, MD., Department of Surgical Gastroenterology, Herlev Hospital, DK-2730 Herlev, Denmark.

concentration of bile acid constituents in patients with external biliary drainage.

### MATERIALS AND METHODS

Seven patients (five women and two men) with total occlusion of the common bile duct due to non resectable periampullary pancreatic cancer were palliatively treated for severe symptomatic jaundice with external biliary drainage. Catherisation of the common bile duct was performed as a part of the diagnostic percutaneous transhepatic cholangiography as described by Burcharth and Nielbo [11]. The volume of the external bile secretion was measured for 24 hour periods during 5–9 days before the day of the investigation at which time the amount of bile secreted per day had become constant. All patients were studied after informed consent and approval by the local Ethics Commitee, and the study was conducted in accordance with Helsinki Declaration II.

The patients were on oral nutrition. The median age was 73 years (range 52–77 years) and the median weight was 56 kg (range 33.4–72.5 kg). Each day of investigation started at 8 am. Somatostatin-14 (DuraScan, Odense, Denmark) was given as a continous 24 hour intravenous infusion (250 μg/hour) by means of a perfusion pump. The infusion rate corresponded to a dosage of 3.5–7.1 μg/h per kg body weight (median 4.5 μg/h per kg BW). The bile was sampled during the last hour before the start of infusion and after the first, second and the last hour of infusion. The total amount of bile secreted during the 24 hour infusion period was measured. Bile samples were taken in dry tubes and frozen to – 20°C until analysis. The bile was analysed for concentrations of cholesterol, triglyceride, $Na^+$, $K^+$, $Cl^-$, $Ca^{++}$, $Mg^{++}$ and bile acids. The blood glucose level was measured four times during the 24 hour infusion period.

### *Assays*

Bile acids were assayed using a HPLC unit (model Hitachi-Merck) equipped with fluorescent detection and Lichrocart 250-4 (Merck-cat. 16056) column. The isolation and determination of free and glycine conjugated bile acids in the bile was performed as described by Kamada et al. [12]. We used free and conjugated bile acids from Steraloids Inc., USA, dicyclohexyl-18-crown-6-ether from Merck, 1-bromacetylpyrene from Wako Chemicals Gmbh, Germany, and cholylglycine hydrolase from SIGMA, USA. The anion exchanger was diethylaminohydroxypropyl Sephadex LH-20 (lipidex DEAP) from Pachard Instruments, The Netherlands, and Bond Elut C18 from Analytical International, USA. Solvents were of Lichrosolv Grade. Taurine conjugated bile acid was purified by a method used by Tangermann *et al.*[13] with the following modifications: after isolation of the taurine conjugated bile acids, these were hydrolysed and the free bile acids converted into fluorescent compounds according to Kamada *et al.* [12]. This separation makes it possible to measure the different fractions of bile acids.

Biliary sodium and potassium concentrations were determined by flame photometry; biliary chloride by titration, and magnesium and calcium by means of atomic absorption photometry. The biliary cholesterol and triglycerides were analysed with monotests and peridochrom, respectively (BOERINGER Mannheim, Germany).

### STATISTICAL METHODS

Friedman test was used for comparison of the bile acid constituents before and during infusion. When Friedman test showed significant values, we used Student-Newman-Keuls method for pairwise multiple comparisons of significant differences (SigmaStat 1.0 Jandel Scientifics GmbH, Erkrath, Germany). All results are given as median values. The level of statistical significance was set at $p < 0.05$ (two-sided).

### RESULTS

Somatostatin infusion reduced the daily bile loss in all seven patients from a median of 473 ml to 140 ml ($p = 0.01$, Figure 1). The median relative reduction in the daily loss of bile was 41% ($p = 0.01$). The reduction in bile secretion was seen from the first hour of infusion and the effect seemed to increase during the infusion period to about one third of the initial volume in the twentyfourth hour of infusion (Table 1). The reduction in bile secretion was uncorrelated with the dosage infused per kg body weight (Figure 1).

There were no significant changes in the concentrations of the measured bile electrolytes but as the secreted bile volume was markedly reduced, hence the daily loss of electrolytes was significantly reduced, too. The most important effect concerned the loss of sodium and chloride, as both were reduced with more than 2 mmol/h corresponding to a median reduction in the daily loss of about 50 mmol (Table 1). There was

**Table 1** Median hourly molar loss of bile constituents before (–1 to 0), and during somatostatin infusion. The p-values are results of Friedman test.

| *constituents* | | *period (hours)* | | | | |
|---|---|---|---|---|---|---|
| | | *–1 to 0* | *0 to 1* | *1 to 2* | *23 to 24* | *p* |
| Cholesterol | (µmol/h) | 20.40 | 14.14 | 11.18 | 15.10 | 0.003 |
| Triglycerid | (µmol/h) | 10.46 | 8.54 | 6.96 | 7.90 | 0.041 |
| $Na^+$ | (mmol/h) | 2.91 | 1.97 | 1.60 | 0.99 | 0.011 |
| $Cl^-$ | (mmol/h) | 2.69 | 1.52 | 1.34 | 0.73 | 0.005 |
| $K^+$ | (µmol/h) | 103.5 | 57.00 | 45.60 | 35.10 | 0.017 |
| $Ca^{++}$ | (µmol/h) | 32.29 | 17.10 | 15.45 | 9.25 | 0.006 |
| $Mg^{++}$ | (µmol/h) | 13.64 | 8.10 | 7.80 | 3.71 | 0.035 |
| Bile volume | (ml/h) | 20.50 | 14.00 | 12.00 | 6.50 | 0.007 |

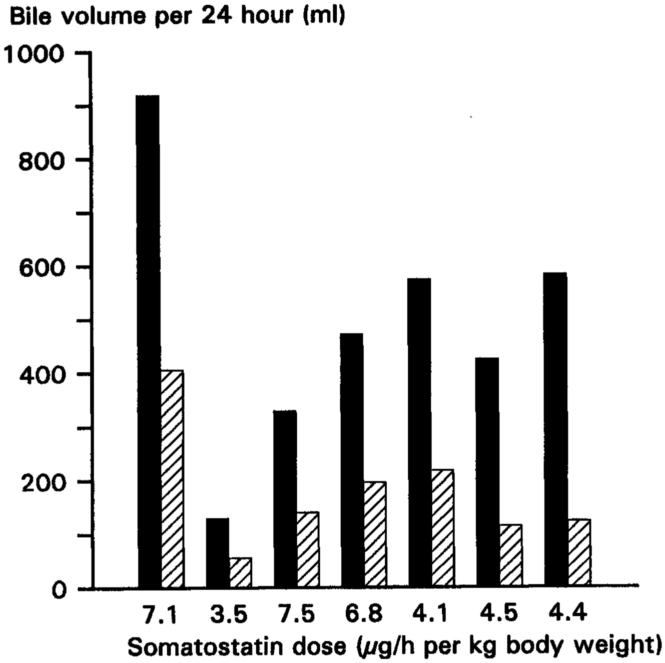

**Figure 1** The diurnal bile secretion before (black columns) and during somatostatin infusion (grey columns) in seven patients arranged in ascending order with the largest relative reduction to the right. The numbers on the abscisse under each patient shows the dosage of somatostatin infused per hour per kg body weight.

also a significant reduction in the molar loss of cholesterol and triglycerid (Table 1).

The significant increase in the concentration of bile acids was mainly due to an increase of glycochenodeoxycholic acid (p = 0.04) giving a significant increase in the glycine conjugated acids (p = 0.01, Table 2). The total hourly molar loss of bile acids showed a marginally significant reduction (p = 0.051, Table 3). The molar loss of taurocholic acids decreased significantly (p = 0.035) giving a significantly decreased loss in taurine conjugated acids (p = 0.035). Also the molar loss of chenodeoxycholic acids decreased significantly (p = 0.046). The marginally significant reduction in total molar bile loss can be explained by a corresponding increased concentration of glycochenodeoxycholic acids. The ratio between the cholic acids and the chenodeoxycholic acids did not change (p = 0.89).

The levels of significant differences, revealed by multiple comparison, were all found between the value before start of infusion and one or several values during infusion.

Serum bilirubin levels were unchanged during the investigation and no side effects due to somatostatin were experienced. Blood glucose remained within the normal range during the 24 hours of infusion.

**Table 2** The median molar concentrations of bile acids before (–1 to 0), and during somatostatin infusion. The p-values are results of Friedman test.

| *constituents* | | *period (hours)* | | | | |
|---|---|---|---|---|---|---|
| | | *–1 to 0* | *0 to 1* | *1 to 2* | *23 to 24* | *p* |
| Glycocholic acid | (mmol/l) | 1.25 | 0.70 | 0.90 | 2.00 | 0.08 |
| Glycochenodeoxycholic acids | (mmol/l) | 0.60 | 0.60 | 0.70 | 1.00 | 0.04 |
| Taurocholic acid | (mmol/l) | 1.25 | 0.75 | 0.80 | 1.10 | 0.22 |
| Taurochenodeoxycholic acids | (mmol/l) | 2.50 | 2.05 | 2.50 | 2.90 | 0.51 |
| Glycine conjugated acids | (mmol/l) | 2.60 | 1.65 | 1.90 | 5.00 | 0.01 |
| Taurine conjugated acids | (mmol/l) | 4.00 | 3.20 | 3.30 | 5.70 | 0.23 |
| Cholic acids | (mmol/l) | 2.50 | 1.40 | 1.40 | 3.10 | 0.10 |
| Chenodeoxycholic acids | (mmol/l) | 3.30 | 2.50 | 3.00 | 5.10 | 0.19 |
| Median of sum of bile acids | (mmol/l) | 6.60 | 5.50 | 5.20 | 8.50 | 0.02 |

**Table 3** Median hourly molar loss of bile acids before (–1 to 0), and during somatostatin infusion. The p-values are results of Friedman test.

| *constituents* | | *period (hours)* | | | | |
|---|---|---|---|---|---|---|
| | | *–1 to 0* | *0 to 1* | *1 to 2* | *23 to 24* | *p* |
| Glycocholic acid | (µmol/h) | 25.63 | 12.00 | 7.50 | 26.00 | 0.53 |
| Glycochenodeoxycholic acids | (µmol/h) | 13.60 | 9.00 | 6.50 | 5.85 | 0.37 |
| Taurocholic acid | (µmol/h) | 25.63 | 12.00 | 12.50 | 14.30 | 0.035 |
| Taurochenodeoxycholic acids | (µmol/h) | 60.00 | 23.58 | 32.50 | 26.00 | 0.08 |
| Glycine conjugated acids | (µmol/h) | 45.10 | 19.60 | 12.00 | 32.50 | 0.44 |
| Taurine conjugated acids | (µmol/h) | 97.38 | 48.00 | 42.90 | 40.30 | 0.035 |
| Cholic acids | (µmol/h) | 51.25 | 24.00 | 20.00 | 40.30 | 0.18 |
| Chenodeoxycholic acids | (µmol/h) | 78.00 | 30.00 | 39.00 | 37.70 | 0.046 |
| Median of sum of bile acids* | (µmol/h) | 142.48 | 77.00 | 54.60 | 79.30 | 0.051 |

*Only one patient had small measurable amounts of free acids. These amounts are included in his sum of bile acids.

### DISCUSSION

Our study demonstrated that the reduction of bile secretion persisted during 24 hour of somatostatin infusion with increasing effect during the infusion time. The present results of the somatostatin effect on bile is in accordance with other studies but in no studies was somatostatin infused for more than three hours [2,8–10]. In dogs, the infused dose needed to be higher than 0.1 µg/h/kg BW to induce a reduction in bile volume[7]. Doses of 8–12 µg/h/kg BW decreased bile flow, while concentrations of cholesterol and bile acids were unchanged [6,7]. Bickerstaff and colleagues infused 48 µg/h/kg BW somatostatin for 20 minutes in dogs and found a decrease in bile flow in accordance with other studies [14]. They recorded slightly increased concentrations of bile acids without definite changes in bile acid output despite the high doses infused [14]. In rats, a dose of 20 µg/h/kg BW caused an immediate decrease in bile flow and in bile acid output to 30–47%[5], while infusion of 500 µg/h/kg BW only reduced the bile output by 20 % [15]. Schirmer *et al.* demonstrated that somatostatin had no effect on isolated perfused rat liver supporting the theory that the effect of somatostatin on the liver is primary indirect[15].

We used a median dose of 4.5 µg/h/kg BW which is the manufacturer's recommended dosage. However, full effect can be obtained at lower dosages as illustrated by the patient with the lowest dosage of 3.5 µg/h/kg BW who had the second largest relative reduction in bile flow of 43 % from 130 ml/24h to 56 ml/24h (Figure1).

Somatostatin used in the present dosage seems to influence both the bile acid dependent and the bile acid independent canalicular flow as only the concentration of glycocholic acid increased while that of taurine conjugated acids was unchanged.

However, the present results can not exclude that somatostatin also influences the ductular secretion. Further investigations are needed to reveal the exact regulatory mechanisms of bile acid secretion.

In patients with inoperable malignant biliary obstruction internal biliary drainage by insertion of an endoprosthesis may be used as a palliative treatment, but in some patients only external biliary drainage can be performed. It is well known that enhanced choleresis occurs after relief of biliary obstruction and in some patients with external biliary drainage the volume and daily loss of bile constituents remain at quite high levels resulting in a daily need for correction of electrolytes and fluids.

Continous somatostatin infusion will probably not become the treatment of choice in all patients with external bile drainage. However, this treatment may be used for the few bile depleted patients who have a devastating daily bile loss resulting in a disordered electrolyte and acidity status requiring daily corrections. Somatostatin infusion may also be used for the first few days after insertion of a percutaneous transhepatic biliary tube or after insertion of an endoprothesis, as the decrease in bile secretion may assist in preventing bile leakage. The analysis of changes in bile acid constituents by the HPLC method appears suitable for further investigations of the regulatory mechanisms of bile acid secretion.

### ACKNOWLEDGEMENT

We thank Helma Furhauge and Birgitte Hansen for technical assistance.

### REFERENCES

1. Gyr,K.E. and Meier, R. (1993) Pharmacodynamic Effects of Sandostatin[R] in the Gastrointestinal Tract. *Digestion*, **54**: (suppl. 1), 14–19.
2. Nyberg, B., Angelin, B. and Einarsson, K. (1992) Somatostatin does not block the effect of vasoactive intestinal peptide on bile secretion in man. *Eur. J. Clin. Invest.*, **22**: 60–66.
3. Calhoun, P. and Hanks, J.B. (1984) Hormonal contribution to biliary secretion. *Surg. Gastroenterol.*, **3**: 8–16.
4. Magnusson, I. (1984) Anticholeretic effects of substance P and somatostatin. *Acta Chir. Scand.*, Suppl 521.
5. Ricci, G.L. and Fevery, J. (1981) Cholestatic action of somatostatin in the rat: effect on the different fractions of bile secretion. *Gastroenterology*, **81**: 552–562.
6. René, E., Danzinger, R.G., Hofmann, A.F. and Nakagaki, M. (1983) Pharmacological effect of somatostatin on bile formation in the dog. *Gastroenterology*, **84**: 120–129.
7. Hanks, J.B., Kortz, W.J., Andersen, D.K. and Jones, R.S. (1983) Somatostatin suppression of canine fasting bile secretion. *Gastroenterology*, **84**, 130–137.
8. Nyberg, B. (1990) Bile secretion in man. The effects of somatostatin, vasoactive intestinal peptide and secretin. *Acta Chir. Scand.*, Suppl. 557.
9. Magnusson, I., Einarsson, K., Angelin, B., Nyberg, B., Bergström K. and Thulin, L. (1989) Effect of somatostatin on hepatic bile formation. *Gastroenterology*, **96**: 206–212.
10. Marteau P., Chrétien, Y., Calmus, Y., Parc, R. and Poupon, R. (1989) Pharmacological effect of somatostatin on bile secretion in man. *Digestion*, **42**: 16–21.
11. Burcharth, F. and Nielbo, N. (1976) Percutaneous transhepatic cholangiography with selective catherization of the common bile duct. *Am. J. Roentgenol.*, **127**: 409–412.
12. Kamada, S., Maeda, M. and Tsuji, A. (1983) Fluorescence high-performance liquid chromatographic determination of free and conjugated bile acids in serum and bile using 1-bromoacetylpyrene as a pre-labeling reagent. *J. of Chromatography*, **272**: 29–41.
13. Tangermann, A., Van Schaik, A. and Van der Hock, E.W. (1986) Analysis of conjugated and unconjugated bile acids in serum and jejunal fluid of normal subjects. *Clin. Chem. Acta.*, **159**: 123–132.
14. Bickerstaff, K.I., Garberoglio, C.A., Baker, A.L. and Moosa, A.R. (1983) Hormonal control of biliary lipid secretion in dogs. *Ann. Surg.*, **198**: 168–171.
15. Schirmer, B.D., Kortz, W.J., Miller, R.J. and Jones, R.S. (1985) Is somatostatin a directly acting cholestatic hormone? *J. Surg. Research*. **39**: 237–245.

